# Trends in Corneal Transplantation and Characteristics of Donors in the Chongqing Eye Bank, China: A Retrospective Study, 1999–2018

**DOI:** 10.3389/fmed.2021.750898

**Published:** 2021-10-28

**Authors:** Handan Tan, Meng Lin, Qingqing Gou, Aijia Li, Fengjuan Gu, Quan Liu, Qi Zhang, Mei Xu, Aize Kijlstra, Peizeng Yang, Hong Li

**Affiliations:** ^1^Chongqing Key Lab of Ophthalmology, Chongqing Branch of National Clinical Research Center for Ocular Diseases, The First Affiliated Hospital of Chongqing Medical University, Chongqing Eye Institute, Chongqing, China; ^2^University Eye Clinic Maastricht, Maastricht, Netherlands

**Keywords:** corneal transplantation, eye bank, a retrospective study, donor characteristics, transplantation

## Abstract

**Aim:** This study aimed to analyze corneal transplantation trends and voluntary donor characteristics at the Chongqing Eye Bank in China.

**Methods:** We retrospectively reviewed and analyzed data from January 1, 1999, to December 31, 2018, covering 5,397 preregistered voluntary donors, 1,955 actual donors, 3,910 donated tissues, and 2,374 corneal transplantations.

**Results:** The 5,397 preregistered donors included 13 ethnic groups, with an overall mean age of 39.6 years (SD 21.5) and 3,010 were women (55.8%). The most prevalent education level was college and above (2,546, 47.2%), and the most common ethnic group was Han (5,335, 98.85%). Of the 1,955 actual donors, the male-to-female ratio was 3.3, and the mean age was 57.1 (SD 23.0 years). Based on population size in 2018, Jiangbei county was the most active in donation willingness, with ~60 × 10^−6^ per capita, and the Yuzhong county was the most active in cornea donations, with ~451 × 0^−6^ per capita. Of the 3,910 donated corneas, 2,540 (65.0%) were clinically used. Of those not used, 978 (71.4%) were rejected for poor corneal quality. The 2,374 (93.5%) corneal transplantation procedures were done at the Department of Ophthalmology of the First Affiliated Hospital of Chongqing Medical University and the rest (*n* = 166, 6.5%) were performed in other centers. Of those 2,374 corneal transplantations, there were 1,671 penetrating keratoplasty (70.39%), 700 anterior lamellar keratoplasty (29.49%), and three corneal endothelial transplantations in our center (0.13%). The number of annual corneal transplantations increased by nearly 10 times, from 35 cases in 1999 to 327 cases in 2018. Among them, cases of penetrating keratoplasty and anterior lamellar keratoplasty increased from 27, and eight cases in 1999 to 230 and 94 cases in 2018, respectively. Infectious keratitis (37.0%) was the leading indication for keratoplasty, followed by corneal scar (19.8%). Over the study period, corneal scars dropped from the first (41.1% in 1999–2003) to the second indication (20.5% in 2014–2018), while infectious keratitis advanced to take the lead, ranging from 12.2% in 1999–2003 to 26.3% in 2014–2018.

**Conclusion:** Our study reports corneal donation and transplantation trends in Chongqing over 20 years, showing that infectious keratitis is a leading indication for keratoplasty and that penetrating keratoplasty and anterior lamellar keratoplasty show upward trends. The analysis further suggests that a potential preregistered cornea donor is a female Han, with a higher education level.

## Introduction

Corneal blindness is the third most prevalent cause of visual impairment worldwide, after cataract and glaucoma (1). It has been estimated that, globally, there were 10 million people with bilateral corneal blindness in 2010 (2). A double-sampling epidemiological study of disabled people in China found that there were ~4–5 million people with corneal blindness in one or both eyes (3). The etiology of corneal blindness is complex and varied, with infectious and nutritional corneal diseases leading the list. These diseases are common in developing countries in Asia and Africa (4).

Once a disease affects the corneal clarity, corneal transplantation is the main and currently the only widely accepted treatment for restoration of vision (5). The techniques and indications of corneal transplantation have changed considerably over the years. Surgical techniques have evolved significantly in the past 10 years, and most corneal transplantation centers have reported an increase in the use of the endothelial keratoplasty technique (6–13). However, the rates of technology adoption differ significantly between countries, possibly because of differences in corneal transplantation indications (14). The number of corneal transplantations in most Asian countries is currently lower than in other countries (15). In China, the general practice with regard to corneal transplantation has only been reported for the north of the country (16, 17).

Corneal transplantation depends on the availability of donated corneal tissue, a major limiting factor worldwide (14). Compared to other countries, China has a lower attainment rate because of the lack of awareness and willingness to donate corneas and there is now a long waiting list of patients necessitating a donor cornea (18). Several studies have shown that differences among locations, cultural backgrounds, and emotional items may affect the willingness to donate corneal tissue (18–20). As mentioned above, the information on the situation concerning cornea donation in China has only scarcely been reported and a further analysis was, therefore, the subject of the study presented here.

In this study, we aimed to identify the corneal transplantation trends and the characteristics of donors handled by the Chongqing Eye Bank over a time period of almost 20 years. Insight into these trends may be helpful to increase the future donation rate.

## Materials and Methods

This study was approved by the institutional review board of the First Affiliated Hospital of Chongqing Medical University. The institutional review board granted a waiver from the need for informed consent because of the retrospective nature of the study and since all the data were anonymous. None of the cornea donations mentioned were obtained from executed prisoners.

Data on voluntary donors and donated corneal tissues were retrieved from the records of Chongqing Eye Bank, one of the largest eye banks in China. Between January 1999 and December 2018, the eye bank had 8,023 preregistered voluntary donors and 1,955 actual donors and had supplied 3,910 corneal tissues to various ophthalmology centers in Chongqing. Preregistered voluntary donors refer to those who are willing to donate their cornea and who filled out a voluntary eye donation application form. All copies of the forms filled out by the preregistered donors from 1999 to 2018 were collected from the Chongqing Eye Bank database. Preregistered donations were mainly made by hospice care centers and hospitals. Staff members of the eye bank sometimes were engaged in on-site publicity and propagated information through various media channels. We extracted the basic demographic information in donation application forms, including name, registration age, gender, ethnicity, family address, occupation, education level, and contact information (phone number; executor: the person responsible for contacting the eye bank when the donor dies; the relationship between the executor and the donor). After excluding 2,626 preregistered donors because of incomplete data in the donation application forms, 5,397 preregistered donors were included in this study. A preregistered donor becomes an actual donor after they die, but final consent is needed from family members. According to the current Chinese regulations, a family member can veto the actual donation.

Information was registered for these actual donors, including demographics (gender, age, and area), donor cause of death, death-to-preservation interval (<6 h in summer, <12 h in winter), and preservation methods. The preservation methods of the cornea mainly include fresh preservation and inactive preservation. The former mainly includes mid-term preservation and wet room preservation, while the latter is mainly cryopreservation. Mid-term preservation is defined as placing the cut corneal slices in a preservation bottle containing a mid-term preservation solution and storing in a constant temperature refrigerator at 4°C (no more than 15 days). Wet room preservation and cryopreservation storage are defined as the preservation of complete sterile donated eyeballs in an airtight preservation bottle with saline gauze inside, cornea facing up, and preservation in a constant temperature refrigerator at 4°C (no more than 48 h) and at −80°C (long-term preservation), respectively. Of 1,955 actual donors, all had complete data. Most donors had died from a malignant tumor, followed by cardiovascular and cerebrovascular diseases, accidents, lung disease, and natural death. For the selection of corneal tissues from actual donors, we strictly followed the policies formulated by the National Health Commission of the People's Republic of China (http://www.nhc.gov.cn/). If the corneal tissue had any quality problems, it could not be used clinically. Poor corneal quality mainly included the following: corneal degeneration (including cornea arcus senilis), corneal opacity, metastatic cancers (general cancer metastasizes to the eye), or when the corneal epithelium had suffered from dryness, punctate lesions, and scratches due to corneal exposure or long storage time (refer to details in *Eye bank Quality Management and Control Indicators* and *Eye Bank Operation Technical Guide*, http://www.nhc.gov.cn/). Corneas with an endothelial cell count below 2,000 mm^2^ were not used for penetrating transplantation. A blood sample (6–8 ml venous blood) was taken to check donors for the presence of infectious disease (HIV, syphilis, hepatitis C, hepatitis B, bacteria, fungi, cytomegalovirus (CMV), and others; refer to *Eye Bank Operation Technical Guide* for details). Of these 3,910 corneal tissues, 2,540 were used for keratoplasty. Of those not used, 978 (71.4%) were rejected due to poor corneal quality and the remaining corneas were rejected due to an infectious disease (18.1%) or missing blood sample (10.5%) (Table 1).

**Table 1:** Summary of non-utilization of the cornea due to various reasons.

**Reasons for non-utilization (%)**	**1999–2003**	**2004–2008**	**2009–2013**	**2014–2018**	**Total**
Poor corneal quality^a^	118 (79.7)	260 (77.8)	144 (67.3)	456 (67.7)	978 (71.4)
Infectious disease^b^	24 (16.2)	64 (19.2)	30 (14.0)	130 (19.3)	248 (18.1)
Hepatitis B	12 (8.1)	48 (14.4)	18 (8.4)	98 (14.5)	176 (12.8)
Syphilis	2 (1.4)	10 (3.0)	8 (3.7)	24 (3.6)	44 (3.2)
Hepatitis C	4 (2.7)	0 (0)	4 (1.9)	6 (0.9)	14 (1.0)
Hepatitis B + hepatitis C	6 (4.1)	6 (1.8)	0 (0)	2 (0.3)	14 (1.0)
Blood sample missing	6 (4.1)	10 (3.0)	40 (18.7)	88 (13.0)	144 (10.5)
Total	148	334	214	674	1,370

a *Poor corneal quality mainly included the following: corneal degeneration (including cornea arcus senilis), corneal opacity, metastatic cancers (general cancer metastasizes to the eye), or corneal epithelium with dryness, punctate lesions, and scratches due to corneal exposure or long storage time (refer to details in Eye Bank Quality Management and Control Indicators and Eye Bank Operation Technical Guide, http://www.nhc.gov.cn/)*.

b *In this study, no patients with HIV or cytomegalovirus (CMV) donated corneas*.

The corneal transplantation recipients (*n* = 2,374) in this study were all patients visiting the Department of Ophthalmology of the First Affiliated Hospital of Chongqing Medical University. Patients visiting other centers (*n* = 166) were excluded from the analysis. The surgery type and indication were noted, and the frequency of each was calculated.

Descriptive statistics were performed, and graphs of voluntary donor information, changes in transplantation indications, and transplantation methods were made. Statistical analysis was performed using SPSS V.17.0 software (SPSS Inc., Chicago, IL, United States). Continuous data are reported as mean ± SD. Categorical data are reported as percentages. We divided the 20 years into four periods: 1999–2003, 2004–2008, 2009–2013, and 2014–2018. Statistical tests were performed using the χ^2^ test. Differences with a *P* < 0.05 were considered to be statistically significant. A Choropleth map was generated using a Geographic Information System (ArcGIS Version 10 software) to visually examine the geographic differences in the proportion of voluntary donors and the source of donated corneal tissues. The ArcGIS is a system that creates, manages, analyzes, and maps all types of data. The system connects data to a map, integrating location data with all types of descriptive information.

## Results

### Preregistered Voluntary Donors

**Figure 1:**
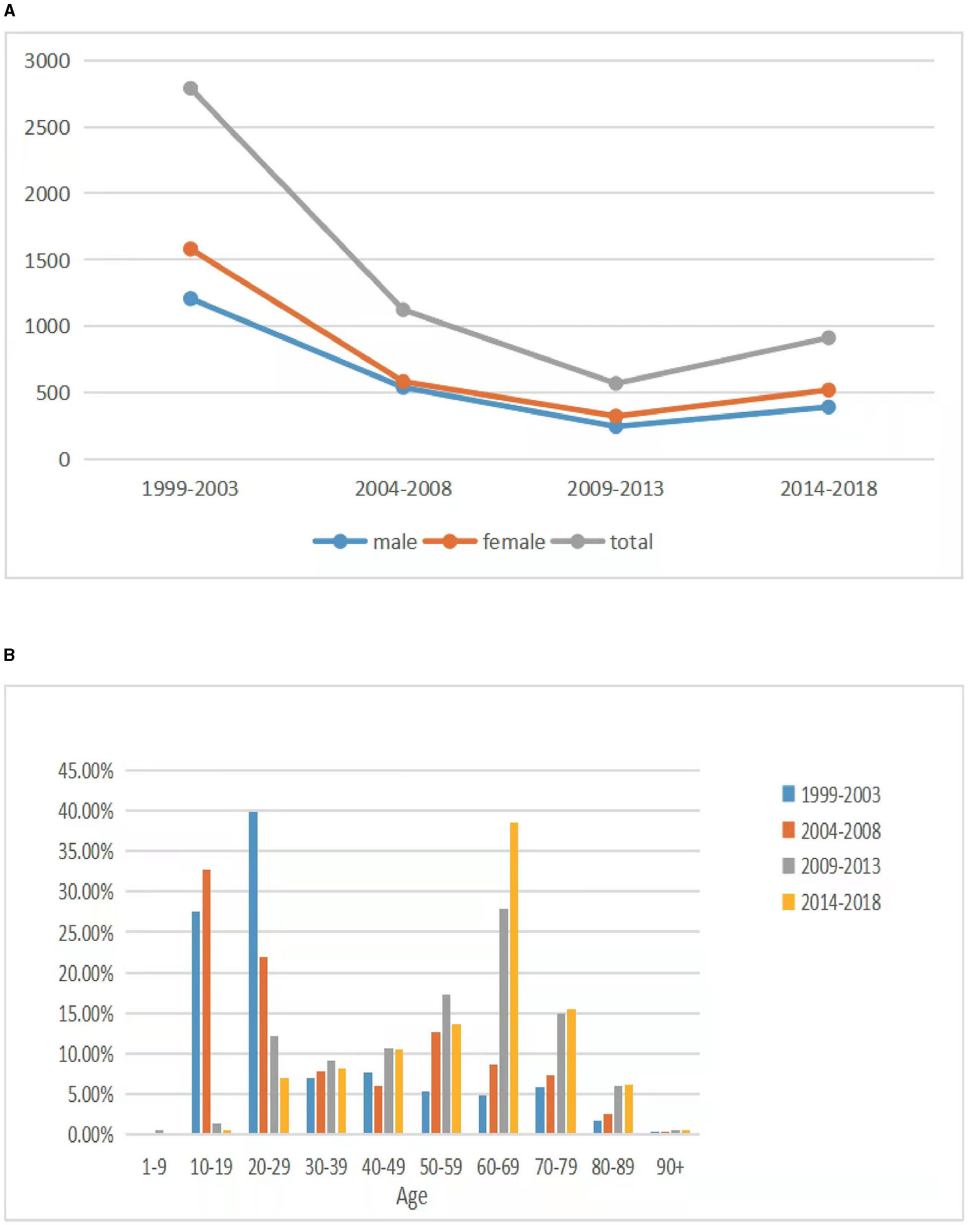
Preregistered voluntary donors in each period. **(A)** The trend in the numbers of preregistered donors and the sex ratio in each period. **(B)** Age distribution of the preregistered donors.

A total number of 5,397 preregistered donors were included in the study. Figure 1A shows the decreasing tendency in the numbers of registrants and the change in the sex ratio during the study period. Among the 5,397 donors, 2,387 were men (44.2%) and 3,010 were women (55.8%). The overall mean age was 39.6 ± 21.5 years; the mean age for male and female donors was 42.0 ± 22.5 and 37.8 ± 20.5 years, respectively. The age distribution of the donors is shown in Figure 1B. Between 1999 and 2003, the most prevalent donor age group was 30–39 years, whereas between 2014 and 2018, it was 60–69 years. The most prevalent education level was college and above (*n* = 2,546, 47.2%), followed by junior college (*n* = 1,062, 19.7%), junior high school (*n* = 862, 16.0%), senior high school (*n* = 553, 10.2%), and primary school and below (*n* = 374, 6.9%).

Except for one man from the Netherlands, all donors were from China. These were members of 13 ethnic groups, including Han (5,335, 98.85%), Tujia (24, 0.44%), Mongolian (12, 0.22%), Miao (5, 0.09%), Manchu (5, 0.09%), Hui (4, 0.07%), Zhuang (3, 0.06%), Yi (2, 0.04%), Yao (2, 0.04%), Chuanqing (2, 0.04%), Dong people (1, 0.02 %), Gelao (1, 0.02%), and Li (1, 0.02%).

**Figure 2:**
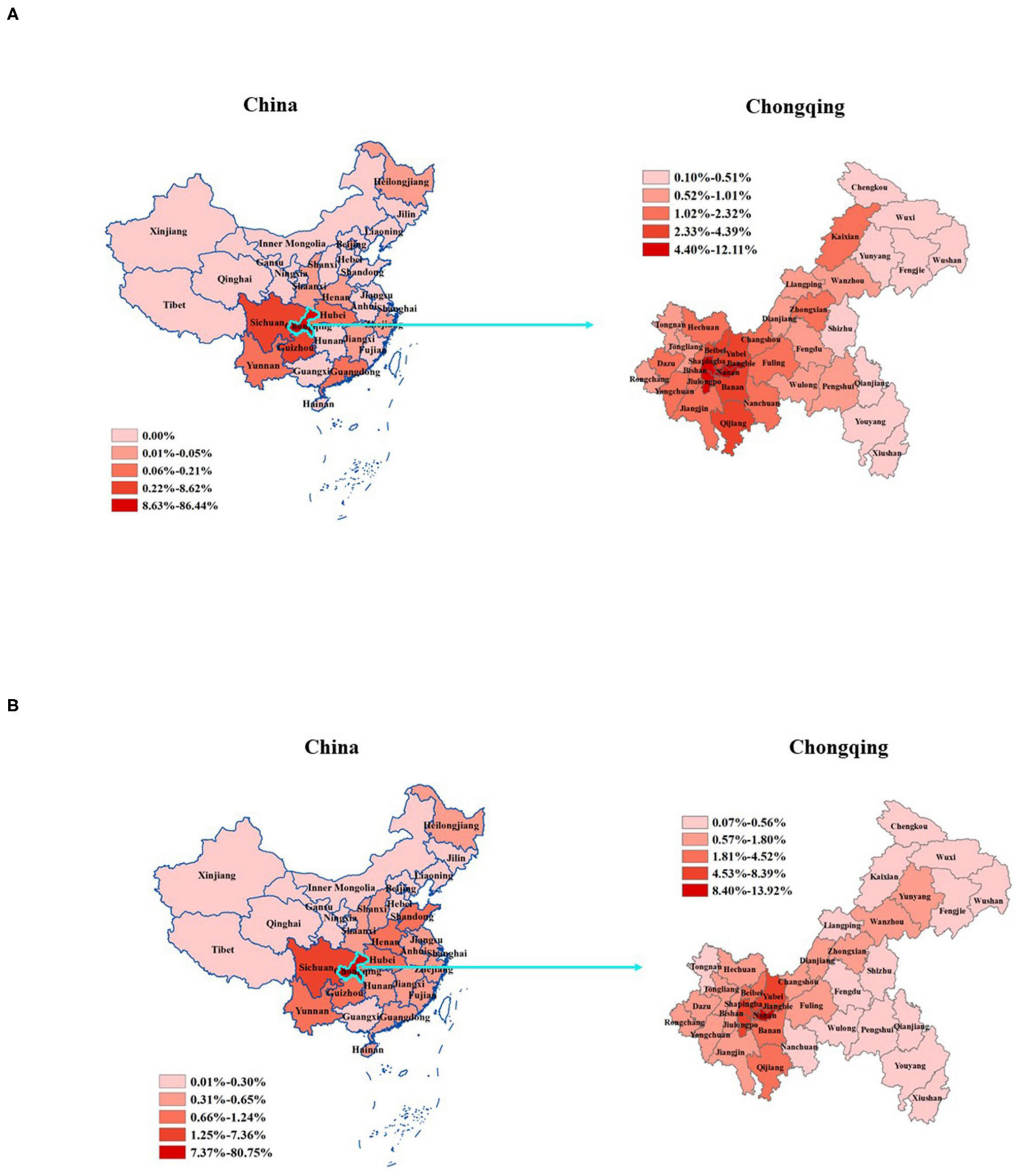
The frequency of corneal donor tissue sources and voluntary donors from different regions of Chongqing and China, 1999–2018. **(A)** The frequency of preregistered voluntary donors. **(B)** The frequency of actual donors. We used quintiles to define map strata. The dark red areas represent higher frequencies.

Quintile-stratified differences in the frequencies of donors at the county level showed that it ranged from 0.07 to 13.93% whereby the Chongqing, Yuzhong, and Nanan counties were in the highest quintile (Figure 2A right panel). Figure 2A left panel illustrates the geographic variation in the distribution of preregistered corneal donors at the province level. Based on the population size in 2018, Jiangbei county (6,14,100 inhabitants and 37 preregistered donors) was the most active in donation willingness, with ~601 × 0^−6^ per capita.

### Eye Banking

**Figure 3:**
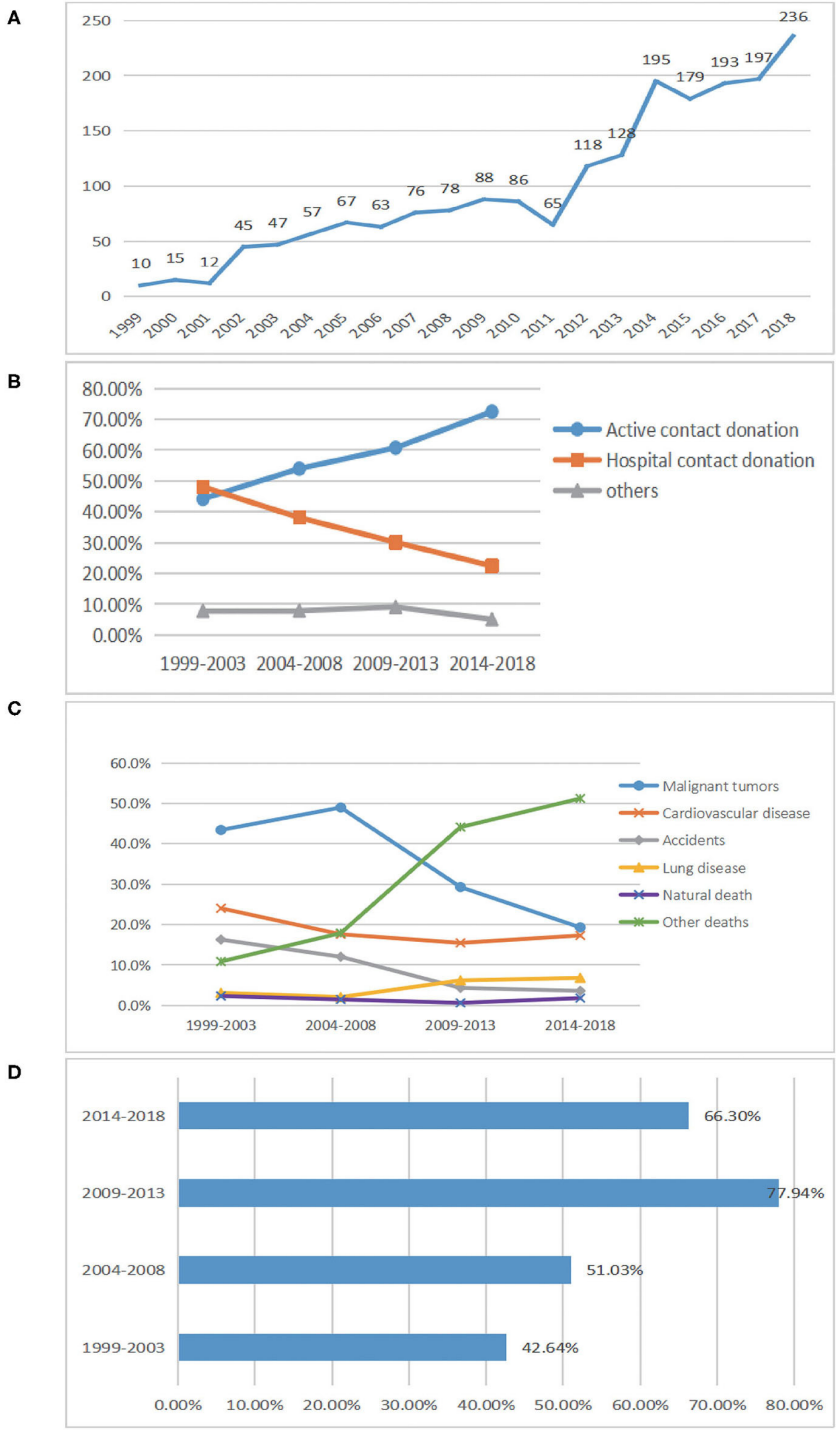
Eye banking from 1999 to 2018. **(A)** The number of actual donations from 1999 to 2018. **(B)** Corneal access channels of the 1,955 actual donors. The corneal access channels included donors or their families who actively contacted the eye bank (active conduct donation), donors or their families that were contacted by the eye bank through hospitals (hospital contact donation), and others (e.g., funeral services and nursing homes). Of these donors, 1,904 (97.4%) were from Chongqing, and 51 (2.6%) came from other regions in China (refer to details in Figure 2B). **(C)** Causes of donor deaths. Accidents such as car accidents, electric shock, drowning, falling from a building, burns, and sudden death are classified as accidental injuries; deaths caused by other diseases, suicide, and unexplained death are classified as others. Lung disease excluded lung cancer. **(D)** The corneal utilization rate in each subperiod. The corneal utilization rate of the 2,540 eventually used for transplantation in each subperiod (1999–2003, 2004–2008, 2009–2013, and 2014–2018).

A total of 1,955 actual donors were analyzed in our study. Of the donors, 1,904 (97.4%) were from Chongqing, and 51 (2.6%) came from other regions in China (Figure 2B). The male-to-female ratio of the actual donors was 3.3 (1,496/459), and the mean age was 57.1 ± 23.0 years. The difference between the gender ratio of the actual donors as compared to that of the preregistered donors is caused by the fact that family members often veto the actual donation of a female preregistered donor. The Chongqing general population slightly increased from about 30.7 million residents in 1999 to ~31.0 million in 2018 (Chongqing Statistics Bureau: http://www.tjcn.org/tjgb/22cq/2682_3.html). The donation rate is, thus, nearly 7 per million residents per year. The annual number of actual donations ranged from 10 to 236 in the time period 1999–2018 (Figure 3A). Based on the population size in 2018, Yuzhong county (5,07,700 inhabitants and 23 actual donors) was the most active in cornea donations, with ~451 × 0^−6^ per capita.

The corneal access channels included donors or their families who actively contacted the eye bank (64.5%), donors or their families that were contacted by the eye bank through hospitals, funeral services, and nursing homes, and others (35.5%). Actual donor numbers showed a gradual growth trend over the years (Figure 3B). In the first time period (1999–2003), malignant tumors (43.4%) were the leading cause of death in our corneal donors, followed by cardiovascular and cerebrovascular diseases (24.0%), accidents (16.3%), lung cancer (13.2%), and deaths caused by other diseases (10.9%). However, the frequency of those who died due to malignant tumors and cardiovascular and cerebrovascular diseases gradually decreased, while the proportion of donors with deaths caused by other diseases obviously increased (Figure 3C).

Between January 1999 and December 2018, 3,910 corneas were donated to the Chongqing Eye Bank. Of these, 2,540 (65.0%) were eventually used for transplantation. The corneal utilization rate in each sub-period is shown in Figure 3D. Of the corneas that were not used clinically, 978 (71.4%) were of poor corneal quality, 248 (18.1%) came from a donor with an infectious disease, and 144 (10.5%), a blood sample was missing (Table 1).

### Corneal Transplantation

**Figure 4:**
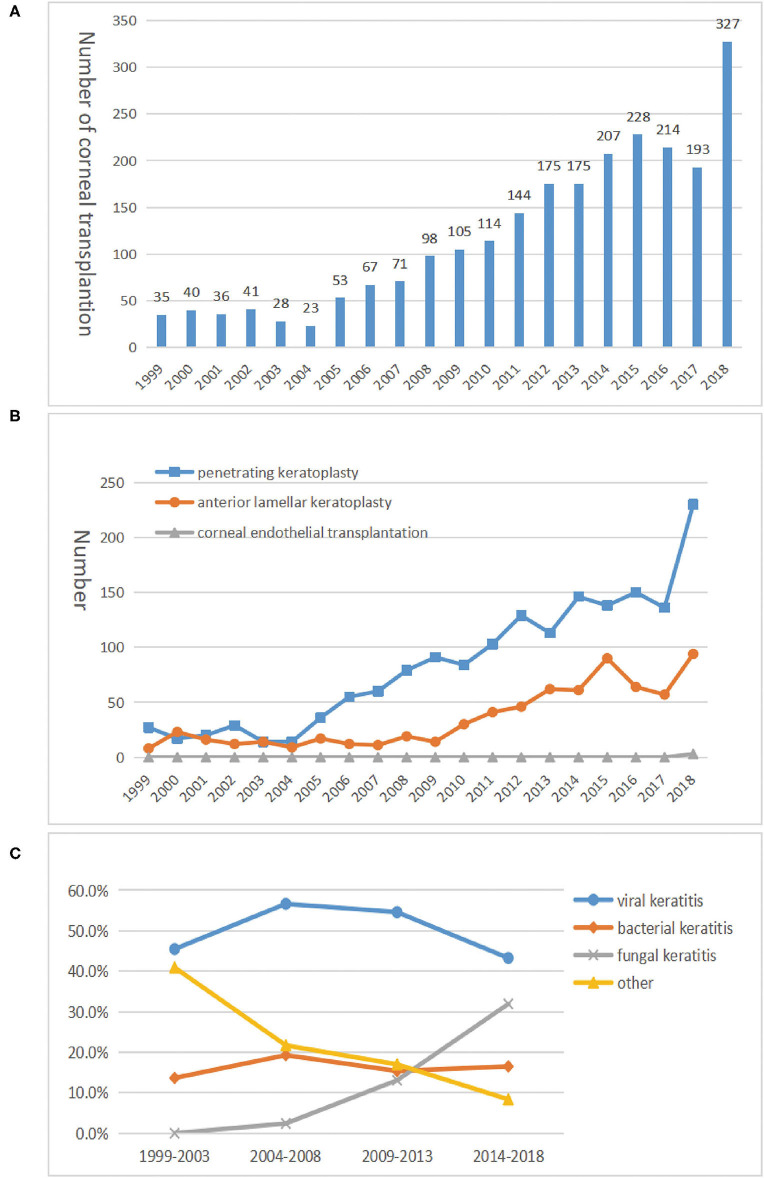
The trend of corneal transplantation from 1999 to 2018. **(A)** The tendency of corneal transplantation numbers. The tendency of 2,540 corneal transplantations showing a significant upward trend from 1999 to 2018. Among these corneal transplantations, 2,374 (93.5%) were done at the Department of Ophthalmology of the First Affiliated Hospital of Chongqing Medical University, and the rest (*n* = 166, 6.5%) were performed in other centers. **(B)** Number of procedures by the three corneal transplantation techniques in each period. Three corneal transplantation techniques indicate penetrating keratoplasty, anterior lamellar keratoplasty, and corneal endothelial transplantation. **(C)** The proportion of infectious keratitis in each period. Infectious keratitis indicated fungal, viral, or bacterial keratitis and others, and they were in the acute stage. Others represent cases where the clinical manifestations are strongly suggestive of an infectious lesion, but the culture results or blood testing were negative.

In total, 2,540 corneal transplantations were performed, of which 2,374 (93.5%) were done at the Department of Ophthalmology of the First Affiliated Hospital of Chongqing Medical University. The rest (*n* = 166, 6.5%) were performed in other centers. During this period, an average of 127 keratoplasties was done each year, ranging between 35 (1.1 per million residents) in 1999 and 327 (10.5 per million residents) in 2018, and the number of corneal transplantations has increased by nearly 10 times (Figure 4A). Of the 2,374 corneal transplants, 1,671 (70.38%) were penetrating keratoplasty, 700 (29.49%) were anterior lamellar keratoplasty, and only three (0.13%) were a corneal endothelial transplantation. Among them, cases of penetrating keratoplasty and anterior lamellar keratoplasty increased from 27 and eight cases in 1999 to 230 and 94 cases in 2018, respectively (Figure 4B).

The indications for corneal transplantation are shown in Table 2. In total, infectious keratitis was the leading indication (*n* = 878, 37.0%), followed by corneal scar (19.8%). Over the study period, corneal scars dropped from the first (41.1% in 1999–2003) to the second indication (20.5% in 2014–2018), while infectious keratitis advanced to take the lead, ranging from 12.2% in 1999–2003 to 26.3% in 2014–2018. Among the infectious keratitis cases, the proportion of fungal causes gradually increased, whereas viral and bacterial causes decreased (Figure 4C). However, a viral etiology was always more prevalent than the non-viral etiologies (*P* = 0.00703).

**Table 2:** Indications for corneal transplantation.

**Indications (%)**	**1999–2003**	**2004–2008**	**2009–2013**	**2014–2018**	**Total**
Infectious keratitis^a^	22 (12.2)	83 (26.6)	389 (42.3)	384 (26.3)	878 (37.0)
Corneal scar	74 (41.1)	93 (29.8)	106 (11.5)	197 (20.5)	470 (19.8)
Repeated transplant	10 (5.6)	28 (9.0)	115 (12.5)	138 (14.3)	291 (12.3)
Corneal trauma	17 (9.4)	48 (15.4)	94 (10.2)	46 (4.8)	205 (8.6)
Keratoconus	13 (7.2)	21 (6.7)	47 (5.1)	47 (4.9)	128 (5.4)
Dermoid tumor	13 (7.2)	8 (2.6)	51 (5.5)	51 (5.3)	123 (5.2)
Bullous keratitis	13 (7.2)	14 (4.5)	38 (4.1)	43 (4.5)	108 (4.5)
Corneal dystrophies	3 (1.7)	15 (4.8)	29 (3.2)	33 (3.4)	80 (3.4)
Mooren's ulcer	3 (1.7)	0 (0.0)	30 (3.3)	12 (1.2)	45 (1.9)
Others	12 (6.7)	2 (0.6)	21 (2.3)	11 (1.1)	46 (1.9)
Total	180	312	920	962	2,374

a *Infectious keratitis indicated fungal, viral–bacterial keratitis, and others, and they were in the acute stage. Others represent cases where the clinical manifestations are strongly suggestive of an infectious lesion, but the results of the culture or blood tests were negative*.

## Discussion

In this study, we found a marked difference in the gender ratio of preregistered cornea donors compared to the gender ratio of the actual donors. Whereas the ratio was similar at the time of registry for donation, almost three times more men became the actual donors compared to women. According to the current Chinese laws and eye bank practices, family members can veto a donation of organs and tissues from deceased relatives. (Decree of the State Council of the People's Republic of China (No. 491st)– human organ transplantation ordinance. Available at: http://www.gov.cn/gongbao/content/2007/content_621229.htm). In other words, if a married man dies, the wife has the right to decide whether to donate the cornea of the husband and vice versa. In practice, we see that husbands often veto the donation of the corneas of their wives. It is unclear why the attitudes toward postmortem cornea donation are so different between husbands and wives in China. It has been shown that the willingness to donate a cornea among members of the German Ophthalmological Society is influenced by gender, but the willingness in a multicenter analysis in Germany was shown not to be affected by gender (20). More research should be performed to gain further insight concerning the male attitude toward organ donation in China and may help to solve this problem and lead to methods to improve final donor acquisition. Although the number of preregistered voluntary donors in the Chongqing Eye Bank markedly decreased over time, the actual number of people donating corneas has gradually increased. The age distribution of preregistered voluntary donors increased with time. In the first study period (1999–2003), the most prevalent donor age group was 30–39 years, whereas in the last study period (2014–2018) this had increased to 60–69 years. This observation is not in line with earlier observations that older age is a practical barrier to corneal donation in Guangzhou, Southern China (19). One explanation is that, in the early stage, staff members of the eye bank were doing on-site publicity and propagated information through various media channels, which is more readily accepted by young people and available to them. However, in the later period, preregistered donations were mainly made by hospice care centers and hospitals. These results suggest that we should enhance our efforts to encourage corneal donation by younger people and men. However, our findings are similar to those reported in an earlier survey in Germany, whereby younger age was found to be associated with a negative attitude toward corneal donation and a positive attitude was correlated with increasing age, which may be explained by the fact that compared with young people, older individuals have had a greater opportunity to reflect on their deaths (20). In particular, we found that people with higher education levels were more willing to donate, which is consistent with many previous reports from other countries and the north of China (17, 21, 22). A higher education level might lead to a better understanding of corneal donation and transplantation, thus fostering acceptance of the idea of organ donation. Inconsistent with these reports, as observed in Germany (20), they did not find differences among different education levels and indicated that the willingness to donate may be counteracted by means of public education. However, this study still suggests that it may be useful to educate young people about transplantation and donation starting with school education. Interestingly, individuals in the Yuzhong and Jiangbei counties, where the economy is developing rapidly and the per capita Gross National Product increased the most during the study period (http://tjj.cq.gov.cn/tjnj/2018/indexch.htm), were more willing to donate corneas compared to other counties, suggesting that economic development may influence corneal donation. This result is in line with reports showing that lower economic levels are a practical obstacle to corneal donation (21, 23). Among participants of different socioeconomic sectors, employees working in medical sectors have a relatively lower willingness to donate their corneas than employees working in non-medical settings (20). More studies are needed to clarify this issue in the future.

Of the 3,910 corneal tissues acquired by our eye bank, 2,540 were finally used for keratoplasty. This utilization rate is similar to that seen in India and the United States (24–26). The leading reason why a donated cornea could not be used in our eye bank was due to poor corneal quality, which is similar to what was recently reported in India (24). The report from India proposed a quality assurance program (QAP) for increasing corneal utilization; such a program would introduce high-level scrutiny of wasted donated tissues, and the authors suggest that such a QAP would improve the corneal utilization rate. Another report from Germany has also found that the implementation of a quality management system and better management of contamination significantly reduces the rate of discarded donor tissue in the eye bank (27). Moreover, technical improvements, such as the application of anterior segment optical coherence tomograph providing a sterile screening method to identify corneal tissues, have further optimized donor selection and increased the utilization rate (28). Cornea arcus senilis was one of the causes of poor corneal quality as well as corneas with an endothelial cell count below 2,000 per mm^2^. Both factors are known to be associated with donor age (27, 29) and as mentioned earlier, the average age of donors was older than 50 years. A previous study has reported a similar result showing that a high donor age (>80 years), compared to younger donors (age <40 years), reduced the final suitability of corneal donor tissue (30). This report further showed that other factors might also influence the suitability and clinical use of donor corneas, such as a history of cataract surgery and sepsis or multi-organ failure. Another important reason why the corneas could not be used was the fact that our donors often had an infectious disease (such as syphilis, hepatitis C, and hepatitis B), which is an exclusion factor for the clinical utilization of the cornea.

At early stages, malignant tumors were the leading cause of death in our corneal donors, followed by cardiovascular and cerebrovascular diseases. Due to the sudden onset of cardiovascular and cerebrovascular diseases, these patients and their families usually lack awareness of the possibility of corneal donation, resulting in a lost opportunity for reaching a large potential donor population. However, patients with malignant tumors often receive more hospice care than those with cardiovascular and cerebrovascular diseases, which may be one of the reasons for improving the rate of corneal donation. During their stay in hospitals or hospice care centers, there is often sufficient time to promote donation knowledge and discussions on donations with these patients and their families so that they can better understand and accept the idea of tissue and organ donations. Donation promotion in patients with cardiovascular and cerebrovascular diseases is more difficult and should receive more attention in the future. However, over the study period, malignant tumors dropped from the first to the second cause, while other death causes took over the first place. One explanation is that more people have been surviving from tumors and other diseases due to improvements in medical technology, while the lack of more attention to mental health may lead to suicides and unexplained deaths (31, 32).

During the study period, the number of keratoplasty procedures increased in our hospital. In addition to population growth, a possible explanation for this trend might be an increase in the number of corneal donations over time, (7, 33) which is due to an increased awareness caused by joint efforts of the media, government, eye banks, and hospitals. However, compared with the developed countries, the per capita rate of corneal transplantation procedures in China is still relatively low, especially the rate of corneal endothelial transplantation (13, 18). We did not carry out corneal endothelial transplantation until 2018, therefore, the proportion of corneal endothelial transplantation was very low. However, the rate of corneal endothelial transplantation is rapidly increasing in the developed countries and has even passed the number of penetrating keratoplasties (13, 18).

Infectious keratitis was the main indication for a corneal transplant in our center, which is consistent with reports from other regions in China (34, 35). This is different from Western countries, where keratoconus is the leading indication for corneal transplantation (4, 7, 36, 37). This may also explain why we mostly performed penetrating keratoplasties (22). Because the incidence of Fuchs' endothelial corneal dystrophy is increasing, posterior lamellar keratoplasties (Descemet membrane endothelial keratoplasties and Descemet stripping automated endothelial keratoplasties) have surpassed penetrating keratoplasties in centers elsewhere (13). In our study, the proportion of fungal cases increased obviously, and treating fungal keratitis was always a challenge. A recent report has found that 5% of fungal keratitis cases without any suspicion of herpes simplex virus (HSV) displayed a positive HSV PCR test and this complicated infection increased the difficulty to manage these patients (21). We believe that one of the reasons why fungal keratitis increased that much (up to 30%) was due to the abuse of glucocorticoids and antibiotics (23). Another reason is that the improvement and the application of confocal microscopy have increased the detection rate of fungal keratitis (29).

This study has several limitations. First, we excluded 2,626 preregistered donors with incomplete data in their donation application forms. This is because preregistered voluntary donors filled out donation application forms by themselves, and these forms did not undergo a stringent review. Second, in our study, there were no patients with HIV or CMV that donated corneas, which may be due to the limitations of the region, or the insufficient popularity of corneal donation. Moreover, the rate of corneal transplantation procedures in our study is still relatively low, especially the rate of corneal endothelial transplantation.

In conclusion, our study investigated the trends in corneal donation and transplantation in the Chongqing region over a time period of 20 years. Infectious keratitis has become the leading indication for corneal transplantation and penetrating keratoplasty is the most commonly used corneal transplantation technique. The characteristics of potential cornea donors show that individuals with a higher education level are more likely to donate and that economic growth may influence the rate of donation. Due to the current national regulations, de facto cornea donation depends on the final agreement of family members and ultimately results in a markedly higher frequency of male donors.

## Data Availability Statement

The original contributions presented in the study are included in the article/supplementary material, further inquiries can be directed to the corresponding authors.

## Ethics Statement

The experimental procedures and research design were conducted in accordance with the tenets of the Declaration of Helsinki and Declaration of Istanbul. The Clinical Research Ethics Committee of the First Affiliated Hospital of Chongqing Medical University approved this study and waived the need for informed consent based on the retrospective nature of the study and anonymization of the data. None of the cornea donations mentioned were obtained from executed prisoners.

## Author Contributions

HL, PY, and HT: conceived and designed the study. ML, QG, AL, QL, FG, QZ, and MX: collected clinical data. HT and ML: analyzed and interpreted the data and wrote the first draft of the study. HL, PY, and AK: reviewed and edited the manuscript. All authors provided a final review and approved the manuscript before submission.

## Funding

This study was support by the National Natural Science Foundation Project (81974131 and 81770913).

## Conflict of Interest

The authors declare that the research was conducted in the absence of any commercial or financial relationships that could be construed as a potential conflict of interest.

## Publisher's Note

All claims expressed in this article are solely those of the authors and do not necessarily represent those of their affiliated organizations, or those of the publisher, the editors and the reviewers. Any product that may be evaluated in this article, or claim that may be made by its manufacturer, is not guaranteed or endorsed by the publisher.
